# Intergenerational transmission of violence and resilience in conflict-affected Burundi: a qualitative study of why some children thrive despite duress

**DOI:** 10.1017/gmh.2017.23

**Published:** 2017-12-20

**Authors:** L. H. Berckmoes, J. T. V. M. de Jong, R. Reis

**Affiliations:** 1 The Netherlands Institute for the Study of Crime and Law Enforcement, Amsterdam, Noord-Holland, The Netherlands; 2 Amsterdam Institute for Social Science Research, University of Amsterdam, Amsterdam, Noord-Holland, The Netherlands; 3 Boston University School of Medicine, Boston, USA; 4 Department of Public Health & Primary Care, Leiden University Medical Center, Leiden, Zuid-Holland, The Netherlands; 5 School of Child and Adolescent Health, The Children's Institute, University of Cape Town, Leiden, Zuid-Holland, South Africa

**Keywords:** Burundi, caregiving, children, intergenerational transmission, resilience

## Abstract

**Background:**

Research suggests that in environments where community conflict and violence are chronic or cyclical, caregiving can impact how children may begin to reproduce violence throughout the various stages of their lives. The aim of this study is to understand how caregiving affects processes of reproducing violence and resilience among children in conflict-affected Burundi.

**Methods:**

We combined a socio-ecological model of child development with a child-actor perspective. We operationalized the core concepts ‘vulnerable household’, ‘resilience’, and ‘caregiving’ iteratively in culturally relevant ways, and put children's experiences at the center of the inquiry. We carried out a comparative case study among 74 purposively sampled vulnerable households in six *collines* in three *communes* in three provinces in the interior of Burundi. Burundian field researchers conducted three consecutive interviews; with the head of the household, the main caregiver, and a child.

**Results:**

Our findings reveal a strong congruence between positive caregiving and resilience among children. Negative caregiving was related to negative social behavior among children. Other resources for resilience appeared to be limited. The overall level of household conditions and embedment in communities attested to a generalized fragile ecological environment.

**Conclusions:**

In conflict-affected socio-ecological environments, caregiving can impact children's functioning and their role in reproducing violence. Interventions that support caregivers in positive caregiving are promising for breaking cyclical violence.

## Introduction

In environments characterized by chronic or cyclical community conflict and violence, children grow up learning about and having to deal with violence from an early age. This can affect how children may reproduce violence throughout the various stages of their lives (Dickson-Gómez, 2002; Dunlap *et al.* 2004; Gorman-Smith *et al.* 2004; Al-Krenawi & Graham, 2012; Lösel & Farrington, 2012; Valentino *et al.* 2012; Richardson & Van Brakle, 2013). Processes fostering or breaking the intergenerational continuity of violence require urgent attention, especially in conflict-affected environments (Cummings *et al.* 2009). This is important also in Burundi, where successive generations of young people have participated in ethnic and political conflict (Lemarchand, 1994; Berckmoes, 2014, 2015). Since Independence in 1962, ethnic and political violence occurred in 1965, 1969, 1972, 1988, 1991, and from 1993 to 2005 when Burundi finally emerged from a 12-year civil war (Lemarchand, 1994; Uvin, 2009). Since April 2015, the country is again enmeshed in political crisis (UN, 2016).

Research has also shown that in environments of chronic or cyclical violence, family dynamics and caregiving can impact children's functioning and their role in reproducing violence (Gorman-Smith & Tolan, 1998; Fergus & Zimmerman, 2005; Goodkind *et al.* 2012; Lösel & Farrington, 2012; Valentino *et al.* 2012; Betancourt *et al.* 2015). In this regard, the positive effects of proximity to parents and other attachment figures in the midst of the war on child development are one of the most enduring findings in the literature (Masten & Narayan, 2012). Furthermore, family cohesion and specific caregiving practices or combinations thereof (Baumrind, 1991; Gorman-Smith *et al.* 2004), such as monitoring, supervision, involvement, and supportive or close parent–child relationships, are found to protect against negative impacts of community violence (Jarrett, 1997; Gorman-Smith & Tolan, 1998; O'Donnell *et al.* 2002; Bailey *et al.* 2006; Cummings *et al.* 2009; Frey *et al.* 2009; Richardson, 2010; Goodkind *et al.* 2012; Lösel & Farrington, 2012; Richardson & Van Brakle, 2013; Janssen *et al.* 2016) and may promote prosocial behavior and positive, civic engagement (Taylor *et al.* 2017; in, Cummings *et al.* 2017). Family conflict, harsh parenting, physical and psychological abuse, and neglect may worsen negative effects of exposure to community violence and contribute to processes that reproduce violence (Lynch & Cicchetti, 1998; Dunlap *et al.* 2004; Lösel & Farrington, 2012; Valentino *et al.* 2012; Palosaari *et al.* 2013; Cummings *et al.* 2016).

At the same time, there is evidence that war-affected violence may transmit to the family level, making families particularly vulnerable to an increased perpetration of violence toward the children (Catani, 2010; Betancourt *et al.* 2015). Literature also reveals that children affected by war are often confronted with secondary stressors in the household and the community, such as poverty, separation from loved ones, and broken social and community relations (Shaw in Cummings *et al.* 2017). Moreover, children's embedment in the community may shape the mental health and functioning of children exposed to political violence. For instance, insecurity about the community (Cummings *et al.* 2016), social disorder within the community (Betancourt *et al.* 2014), and community stigma (Betancourt *et al.* 2015) have been shown to negatively affect children and youth in conflict-affected environments.

Yet, most research on families, caregiving, and the intergenerational transmission of violence took place in European and Northern American contexts. Although research in non-Western environments is slowly increasing (e.g. Rieder & Elbert, 2013; Saile *et al.* 2014; Song *et al.* 2014; Betancourt *et al.* 2015), studies that also critically explore who is involved in caregiving, what caregiving entails, and what defines a good child or good parent, especially in conflict-affected environments in Africa, remain limited. Children's perspectives and strategies also prove to be largely absent; literature focusing on the transmission of violence generally constructs children as passive victims rather than agents contributing to processes reproducing violence or to resilience – despite calls to understand the relation between the individual and the environment as transactional (Cicchetti in Cummings *et al.* 2017).

In this article, we report on a qualitative study with caregivers and children in vulnerable households in Burundi that aimed to understand how caregiving affects children's resilience to reproducing violence in conflict-affected ecological environments. The study formed part of a partnership project between the University of Amsterdam and UNICEF. We departed from a socio-ecological model to child development (Bronfenbrenner, 1979; Cummings *et al.* 2017) in combination with a child-actor perspective (Reis & Dedding, 2004; Reis, 2013). We approached children's socio-ecological environment as primarily composed of different microsystems in which a child interacts directly with significant others. The mesosystem connects different microsystems and further removed are structures which may indirectly influence the conditions in which the child grows up, such as the political organization and the cultural norms regarding caregiving (exo and macrosystem) (Bronfenbrenner, 1979). We included multiple socio-ecological levels in our analysis (cf. Cummings *et al.* 2017), but focused especially on the household (microsystem) and the community (micro and mesosystem). In this paper, we address the main research question: How is resilience among children affected by the way they are cared for in the household? We explored also if caregiving and resilience were associated with different household conditions and the embedment in the community.

## Methodological approach

### Sampling

The study was designed as a qualitative comparative case study. We purposively sampled 120 households at Burundi's administrative levels of province, *commune* (comparable to district) and *colline* (hill or village), and at the level of the household. We included three of 18 provinces (Cibitoke, Muyinga, and Rumonge). In each province, two *collines* in one *commune* were selected (see Table 1). In each of the six *collines*, 20 households were included. Household, in this study, refers to the (group of) persons with whom the child generally resides and who share their economic assets (Demographic and Health Surveys Program, 2016).

**Table 1.** Taxonomy of vulnerable households

Type of category	Vulnerability characteristic	No	Household category	Commune
Composition of household	Absent parent(s)	1	Female-headed household: divorced or abandoned	Gatete, Buhinyuza
		2	Widowed caregiver (F)	Rugombo, Buhinyuza
		3	Widowed caregiver (M)	Buhinyuza
		4	Grandparents as caregivers	Buhinyuza
		5	Unplanned pregnancy leading to school dropout	Buhinyuza
		6	Child out of wedlock	Rugombo
		7	Child-headed household	Buhinyuza
		8	Fostered child	Gatete, Buhinyuza
	Deviant spousal relationships	9	‘Illegal’ marriage/co-habitation	Gatete, Rugombo
		10	Polygamous household	Gatete, Rugombo, Buhinyuza
Livelihood	Unstable livelihoods	11	Extremely poor	Gatete
		12	Homeless family (‘without address’)	Rugombo
		13	Landless/without cultivatable land	Rugombo
		14	Fishermen	Gatete
	Deviant livelihoods	15	Reputed as sorcerer/poisoner	Gatete
		16	Prostitution	Gatete, Rugombo
		17	Imprisoned parent	Buhinyuza
Health problems	Stigmatized illness	18	HIV/AIDS	Rugombo
		19	Physically handicapped caregiver	Rugombo, Buhinyuza
		20	Mental health problems	Gatete, Rugombo, Buhinyuza
	Substance abuse	21	Alcoholic caregiver	Gatete, Rugombo, Buhinyuza
War–politics–ethnicity	Identity related to war/political affiliation	22	Caregiver handicapped by war	Rugombo
		23	Demobilized combatant	Gatete Rugombo
		24	Belongs to political minority	Gatete
		25	Displaced during civil war	Gatete, Rugombo, Buhinyuza
		26	Returnee after war	Gatete, Rugombo, Buhinyuza
		27	Ascribed identity to ‘those who stayed’	Buhinyuza
	Marginalized	28	Baterambere (indigenous community)	Gatete, Buhinyuza

The three provinces were purposefully selected in consultation with local partners and experts at non-governmental organizations. The communes and *collines* were selected in consultation with the local administration and child protection organizations operating in the three provinces. The leading criteria were that the localities were reputed to be especially vulnerable to tensions and violence related to the past and/or ongoing political contestation. This was important because of our aim to, at a later stage, translate research findings into peacebuilding interventions. Furthermore, we selected three provinces to create geographical spread and variation in the types of challenges faced by households. Although cultural and linguistic characteristics are shared across Burundi (Ntahombaye & Nduwayo, 2007), local conditions vary (Newbury, 2001). Rumonge, situated in the south-west, is home to a substantial Swahili-speaking community. In the north-west, Cibitoke's proximity to Rwanda and Congo influences population and trade; and the altitude and climate of Muyinga, in the north-east of the country, affects livelihood possibilities.

In the *collines*, local authorities and other community representatives helped construct a local taxonomy of 15–20 household categories deemed ‘vulnerable to violence’ according to the local perceptions. The local taxonomies helped us identify, in total, 28 categories of households which can be organized into four groups (Table 1). For each *colline*, out of these 28 categories of households, 10 relevant categories were selected. Selection of 10 locally salient vulnerable household categories and household sampling was done by the off-site supervisory team based in Burundi's capital Bujumbura to reduce selection bias. The selection was guided by our interest in structural vulnerability characteristics (e.g. missing caregiver; chronic health problems) and the criterion that the category was present in at least two *collines*. Households could fit into several categories as multiple vulnerability characteristics can coexist. Per category, we asked local referees to provide a list of four to six households considered to be struggling with various forms of violence in the household or in the community, and a similar list with households they considered doing well. This was derived from our purpose to identify the mechanisms that may explain why some children under duress reproduce violence, while others in similar circumstances do not and show resilience. We sampled one household from each list leading to 20 selected households per *colline*.

### Data collection and interviews

Data collection started end of November 2014 and lasted 5 months. Six Burundian field researchers with relevant education (e.g. Bachelor degree in psychology, sociology, and social work) and qualitative research experience were trained. To control for gender sensitivity, to ensure field researchers could help each other in the field and for safety reasons, one man and one woman were assigned to the same *commune*, each to one *colline*; the *colline* being the smallest administrative unit that still allowed for the inclusion of sufficient households.

Data collection took place in three phases representing inquiries at different socio-ecological levels. This phased set-up allowed field researchers to build trust in the community before conducting the most sensitive interviews, which were part of the third phase. The first phase involved general socio-economic community mapping aimed at understanding the local experiences with political violence and the resources available in the community. Sources included written and oral information from local authorities, community representatives (e.g. church leaders) and non-governmental organizations present in the community. Second, semi-structured interviews were held with household-heads of sampled households to determine poverty levels, household composition, whether the household had been displaced, and conflict and violence between spouses (Cummings *et al.* 2009). We also asked about relations with neighbors, participation in solidarity groups and other collectives, victimization, and justice. In the third phase, field researchers interviewed primary caregivers and later, children, using a semi-structured interview guide with open questions as well as observation techniques to triangulate data. The aim was to understand caregiving practices and identify resilience among children. The (primary) caregiver was identified through five questions about daily time investment, in particular, caregiving tasks, e.g. food, health, play, school progress, and discipline (Giani *et al.* 2015). Given the highly variable household structures, the primary caregiver could be the parent, other kin, or non-kin. If more than one child was present in the household, we proposed the interview to a child aged 8–18, who was available and willing to participate – while aiming to include a similar number of boys and girls.

With the primary caregiver, we investigated caregiving through themes identified in parenting literature and a concurrent ethnographic study with caregivers in Burundi's capital. (This ethnographic study was part of the overall research partnership between the University of Amsterdam and UNICEF, see Berckmoes & Reis, 2016.) Our questions concerned the quality of caregiver–child relationships, the experience of parenthood, knowledge about the child's preoccupations and whereabouts, communication with their children, and education and disciplining practices. We also asked if caregivers had different approaches to different children. We formulated open questions to allow for the emergence of locally and culturally relevant variation. Examples of questions posed, are ‘Can you describe to me what gives you most joy in raising your children?’ and ‘Can you tell me the ways in which you show your children what is good and what is bad?’ With children, we asked detailed questions about who takes care of the child's basic needs and how, the household tasks assigned to the child, disciplining, relations with others inside and outside the household, vignettes to discuss exposure to violence inside and outside the household, and experiences with internalizing and externalizing behavior such as acting out. Examples of questions posed are ‘Who makes sure that you have all you need for hygiene and good health?’, ‘Can you name three ways that are used to correct you at home?’, and ‘If you feel unhappy, angry or when you are afraid, what do you usually do?’ For both caregivers and children, we used a tool with smiley emoticons to help visualize the quality of relations in the household. Interviews with caregivers and children lasted between 45 min and 3 h.

Per *colline*, two to four selected households had to be replaced, because households had moved, husband and wife were listed as separate households, or household members were not present. In three cases, children refused the interview for reasons unknown. A sixth month of fieldwork had been planned, but Burundi's deepening political crisis necessitated early withdrawal from the field. Other obstacles also hampered reaching field sites, such as national fuel shortages and flooding. Due to these circumstances, interviews with children took place in 74 of the 120 sampled households (Table 2).

**Table 2.** Overview of completed interviews per province

Phase 1: research location	Phase 2: first interview – household situation	Phase 3a: second interview – primary caregiver	Phase 3b: third interview – child
Gatete, Rumonge	40	39	23
Buhinyuza, Muyinga	40	40	29
Rugombo, Cibitoke	39	39	22
Total completed data sets	119	118	74

### Analytical strategy

Given our interest in resilience, which was investigated mostly through the interview with the child, we decided to limit our in-depth analysis to the 74 completed cases. In line with prevalent strategies for qualitative analysis, the first step consisted of a thematic analysis of the relevant concepts for this study. Of a subsample, the first and last authors analyzed data from 13 and six cases, respectively, and then discussed ambiguities and contradictions in interpretation. We operationalized concepts iteratively to allow for the emergence of locally and culturally relevant social forms, perceptions, and norms. We thus built on literature about caregiving in conflict-affected environments, the mentioned ethnographic study with caregivers in Burundi (Berckmoes & Reis, 2016), as well as observations noted by field researchers and a preliminary screening of interview data on norms, values, and practices.

This led to an analytical framework that distinguished various aspects related to exposure to wartime violence, household conditions, caregiving, children's social behavior, and resources and threats in the community, which, as a second step, was applied to all cases by the first author. To enable within-case and cross-case comparison, evaluations of the concepts of ‘household conditions’, ‘embedment within the community’, ‘caregiving’, and ‘resilience’ were categorized into positive, mixed or mediocre, and negative. Our evaluation was informed by triangulating the household data with information on normative caregiving obtained through the mentioned ethnographic study with caregivers in Burundi (Berckmoes & Reis, 2016), observations noted by field researchers, and the preliminary screening of interviews on caregiving norms, values, and practices. With these labels, associations were identified between caregiving and children's reproduction of violence or resilience.

We operationalized ‘caregiving’ as adapted to the child's life stage and gender (Berckmoes & Reis, 2016; Levine *et al.* 1994). In Burundi, after weaning, usually at around age 2, caregivers start educating their children, mostly through implicit techniques (e.g. stimulating imitation). At primary school-going age, rule-setting and disciplining practices, including corporal disciplining, become prominent and caregivers monitor children's movement outside the household. When children reach adolescence, caregivers are supposed to stay close to their children, provide them with positive role models, and explain implications of bad behavior. They also encourage their children to make friends who will inculcate good behavior, and for girls, instill fear of boys to prevent premarital sex and pregnancy. Corporal disciplining is generally seen as ineffective and no longer possible for fear of reprisals, instead intergenerational dialogue is expected. (Ibid) We evaluated caregiving as positive when caregivers and children described caregiving practices in compliance with these generally accepted norms of caregiving, while separately marking different aspects of caregiving, such as involvement, (harsh) disciplining, abuse, and neglect. We evaluated caregiving practices as negative when generally accepted norms were not fulfilled.

‘Resilience’ in this study was operationalized as children's prosocial behavior. In a general sense, resilience refers to the capacity to bounce back to a normal (positive) equilibrium after exposure to adversity (Masten *et al.* 1990; Rutter, 1990; Luthar *et al.* 2000). Resilience results from interactions between the child's characteristics, motivations, and actions, and the ecological environment in which (s)he is embedded (Liebenberg and Ungar 2009; Tol *et al.* 2013). However, what is considered normal behavior throughout different life stages varies cross-culturally (Greenfield & Cocking, 1994; Levine *et al.* 1994; Levine & New, 2008; Van Mourik *et al.* 2017). In Burundi, children's life stages are marked by their ability to learn and interact with others, activities in and outside the household, schooling, the extent to which children adhere to societal norms, and puberty. From 2 years up, but especially when reaching a school-going age, important developmental goals include not displaying negative emotions, compliance, showing respect, and contributing to the household to reciprocate care and prepare for adulthood. At puberty, girls are expected to perform modesty and boys are expected to start contributing financially (Berckmoes & Reis, 2016). The prosocial behavior of children was conceptualized as answering to these developmental goals, and antisocial behavior as the enactment of non-normative behavior. We looked at and marked separately the (non-violent) resolution of negative emotions, children's acts of support and care for others in and outside the household, withdrawal and isolation, and violent and anti-social behavior. Resilience was explored from the perspectives of both primary caregiver and child.

### Findings

Our findings report primarily on caregiving and children's social behavior. We also describe household conditions and household's embedment in the community, to contextualize and interpret our findings on caregiving and children's social behavior.

### Household conditions and embedment in the community

We found generally fragile household conditions in the communities. Of the 74 households in our sample, 60 households were extremely poor or poor. The remaining 14 households were usually able to deal with contingencies such as a bad harvest or sudden illness, but they often faced other difficulties. In almost half of all households, mention was made of conflict and violence between caregivers. Several children and caregivers attributed this to alcohol abuse, a problem mentioned in roughly one in five households. We found no clear patterns between household conditions and caregiving or children's social behavior.

With regards to embedment in the community, most respondents were positive about relations with neighbors, yet these were commonly explained as the absence of bad relations. In few cases, good relations translated into moral support and encouragement, mediation in fights between spouses, watching over the children when caregivers were away, and visits. Material support appeared largely absent. Some people attributed this to ‘hearts changed’ after the war. Theft, jealousy, discrimination, land conflicts, and tensions between adherents of different political parties were found in all communities, but experience with victimization appeared most extreme in Rumonge. For instance, several respondents there mentioned that their houses had been burnt by community members. Rumonge province has a particularly long history of conflict. Cycles of violence, refuge, and return since 1965 have entrenched contestations over land ownership and social and political divisions there. In all three provinces, the overall security situation was estimated as largely positive by most respondents. Yet as mentioned before, during fieldwork, political tensions in all three provinces rose, with security incidents witnessed by our field researchers, and some inhabitants fled. We found no clear patterns between embedment in the community and caregiving or children's social behavior.

### Caregiving

In this study, 59 primary caregivers were women and 15 were men. One in three households was single-headed. We found that roughly 40% of caregivers emphasized affectionate caregiving. To give an example of affectionate caregiving, the case of a mother of five biological and two adoptive children is informative. The mother explained: ‘[the relationship is] good…if I take a long time to return home, my children ask me why I was away for such long time’. She knows when something is wrong, because ‘they come talk to me, they are not afraid of me’, or ‘because the situation or their mood changes, I follow them all the time’. She is happiest when ‘children come talk to me when they need something and I can easily give it to them’. She expects children to participate in the household chores, but allows time for leisure. The younger children she asks to ‘copy the good behavior of those older than them’. Children who do not listen, are punished, ‘because I love them and to prevent them making the same mistakes again’.

Common caregiving problems were the physical absence of caregivers during large parts of the day, that caregivers were unaware or seemed disinterested in what preoccupied their children, that household members felt that each was left to fend for oneself, that (one of the) children were not being taken care of and harsh disciplining (corporal and emotional). An example of a household that showed all of these difficulties was one composed of a mother abandoned by her husband with four children aged between 5 and 12 years old. The mother's work outside the household affects caregiving substantially: ‘I alone cannot feed and clothe and discipline my children, because I have to work outside and leave the children at the house alone’. She felt that the relationships with her children are bad, because ‘I always give severe punishments’. She knows when something is wrong with the two oldest children when ‘they have a tendency to cooperate with me and show that they will not misbehave again’, and for the youngest two when ‘they stop playing and it seems as if they are cold and tired’. The children do not talk to her, ‘because they are afraid of me’. When she wants her children to do something, she gives strict orders. Her three children who had reached school-going age dropped out. To stimulate the youngest to return, she keeps her ‘busy with household chores and [with no] time to play, [so that] she will tell me ‘I return to school’ because children do not like household chores’. She no longer looks after the two oldest boys, because ‘they will end up in prison’.

Although we found such harsh caregiving to be uncommon, many caregivers failed to take care of their children. One-third of the children interviewed (*n* = 26) described that they were not being taken care of and in more than 10 other households, caregivers appeared lax in providing protection, were not engaged in the lives of (at least one of) their children, or the interviewed children said that they felt unsupported or not loved. We encountered forms of harsh disciplining in roughly 20 out of 74 households. In these households, children reported being constantly insulted, feeling like a prisoner, or having been beaten almost to death. Caregivers and children did not always recognize differences between disciplining and abuse, corporal disciplining being a generally accepted practice and sometimes read as a sign of caring about the future wellbeing of the child, especially for children of school-going age (Berckmoes & Reis, 2016). For instance, when a 14-year-old boy we interviewed made a mistake, the father would tie him to a tree, deprive him of food, or lock him outside for the night. While both the father and son explained these punishments as part of a caring relationship, the mother, the primary caregiver in this household, said that she regularly argued with her husband over these disciplining practices.

### Resilience

Forty-one boys and 33 girls between ages 5 and 20 years (median age 12) were included. In total, one-third of them exhibited prosocial behavior with equal numbers for boys and girls. An illustrative example is a 9-year-old boy in Muyinga. The boy explained that when his mother asked him to, he fulfilled tasks such as getting water from the pump, sweeping the house or cooking simple meals, ‘within the limits of what I am capable of doing’. Sometimes he visited family members in the neighborhood, who would then say, ‘thank God you are here, help me do this or that, God will take you to heaven’, and occasionally he helped with the household chores of his friend next door. When afraid, ‘I go where there are many people’, and when unhappy, he would ‘play with others’.

An example of a child exhibiting antisocial behavior is a 12-year-old boy in Rumonge. The boy explained that his mother asked him to help in the household but he rather invested in his ‘personal work’ because he was alone in making sure he had something to eat each day, felt ‘adult’ (*majeur*) and said that household chores were something for girls. When we asked about negative emotions, he said, ‘when a friend harasses me, I cannot battle with him and I isolate myself from others’. He then narrated the following experience: ‘One day I was playing on the road when a friend insulted me. I did not say anything and went home very frustrated. On the way home, I came across a neighbor's child who did not let me pass first. I threw him on the ground violently’. Almost 20 children exhibited various forms of antisocial behavior, of whom only three were girls. Of the children interviewed in Rumonge, almost half showed antisocial behavior, compared with three and four children in the other provinces. Antisocial behavior was witnessed primarily in relation to how children dealt with negative emotions and in children's interaction with peers. Half of the children portraying antisocial behavior showed respect to and were obeisant of their caregivers. In the roughly 40% of households where conflict or violence between caregivers occurred, one-third of the children displayed antisocial behavior, while another third displayed prosocial behavior.

For one-third of the children, we found inconsistencies in the answers or between interviews with the caregiver and the child. In other cases, findings were inconclusive, such as in the case of a 12-year-old daughter of a man known by our local referee as extremely violent: ‘When [the father] beats [his daughter], you would say he does not recognize her as a human being’. The girl made no mention of violence in the household, which the field researcher interpreted as follows: ‘Either the child has internalized the violence she experiences to the point that she does not recognize it anymore, or she does not want to disclose the household secrets to an unknown person’.

### Case comparisons

We found strong congruence between caregiving and children's social behavior. In almost 85% of the 40% households characterized by affectionate caregiving, children exhibited largely prosocial behavior, and no child displayed mostly antisocial behavior. In almost 75% of the 30% households characterized by harsh or neglectful caregiving, children exhibited antisocial behavior in multiple domains (emotional and behavioral, within and outside the household). We encountered only four children who displayed prosocial behavior despite mediocre or negative caregiving, three of whom mentioned other relational resources, such as a supportive grandmother. The fourth, a 15-year-old girl, reported that converting to another religion had helped her, but that her brothers regularly isolated themselves, yelled at others, fought with peers, and had stolen from neighbors. Strong relations were found between specific harsh or neglectful caregiving practices and children's social behavior. In households where children reported not being taken care of or lacking support or love (in half of the 74 households), only two children exhibited largely prosocial behavior. In the households where mention was made of harsh disciplining, 13 out of 20 children displayed antisocial behavior in multiple domains.

## Discussion

This study departed from a socio-ecological model to child development, including multiple system levels (Bronfenbrenner, 1979; Cummings *et al.* 2017). It aimed to identify and understand whether and how caregiving may affect resilience among children in conflict-affected environments, which in this study was operationalized as children's social behavior in the household and the community in Burundi. Furthermore, it explored whether household conditions and embedment in the community affected caregiving and children's social behavior.

In line with existing literature (e.g. Jarrett, 1997; Gorman-Smith & Tolan, 1998; O'Donnell *et al.* 2002; Bailey *et al.* 2006; Cummings *et al.* 2009; Frey *et al.* 2009; Richardson, 2010; Goodkind *et al.* 2012; Lösel & Farrington, 2012; Richardson & Van Brakle, 2013; Janssen *et al.* 2016; Taylor *et al.* 2017; in, Cummings *et al.* 2017), our findings reveal a strong congruence between affectionate caregiving and children's resilience, suggesting that affectionate caregiving works protectively in conflict-affected socio-ecological environments. Similarly, harsh and neglectful caregiving was strongly associated with children's antisocial behavior, suggesting it is a risk factor affecting resilience. A limited number of children displaying antisocial behavior still displayed normative behavior in the household. This suggests that when things go wrong, children protect relationships within the household longest. We hypothesize that this may be because of the importance attributed to family in Burundian society and because of children's dependence on (biological) caregivers (Berckmoes & Reis, 2016), which may be exacerbated by the generalized fragile community environment. Alternatively, children may fear harsh disciplining at home the most, and therefore display prosocial behavior in the household more than in the community.

In the few cases where children displayed positive social behavior despite mediocre or largely negative caregiving, children appeared to draw on other resources in the relational sphere (Reijer, 2013). In one case, which we described above, the child actively searched for other support structures (religion). The case exemplifies the significance of children's agency in the processes of reproduction of violence or for resilience. Moreover, the fact that this girl's brothers displayed largely negative social behavior is a reminder that not all children are affected in the same way by the same circumstances, and that gender ‘likely plays complex roles in the context of extreme adversity’ (Masten & Narayan, 2012: 240; Cummings *et al.* 2017).

We found no variation in prosocial behavior between boys and girls, but we found variation in antisocial behavior, with boys portraying antisocial behavior more often than girls. This may be because expressions of psychological distress can differ among boys and girls [Bongers *et al.* 2003; Masten & Narayan, 2012; (Betancourt *et al.* in Cummings *et al.* 2017)]. We hypothesize that it may also be related to differences in caregiving toward boys compared with girls. For instance, Berckmoes & Reis' study (2016) among parents in Bujumbura shows that parents perceive raising boys as more challenging than raising girls, and Charak *et al.* found a higher prevalence of physical abuse among boys than girls in Burundi (2017). Differences may also result from transactional effects between caregivers and children (Sameroff & Fiese, 2000).

Notwithstanding the careful attention to (fragile) household conditions and their potential effects on caregiving and children's social behavior, we had limited possibilities for differentiation between households and thus found no clear pattern. This may (partly) result from our sampling frame, but is in line with studies relating the overall devastating effects of the civil war on Burundian society (Reyntjens, 2005; Uvin, 2009; Berckmoes, 2014). Our study revealed a generalized fragile ecological environment, suggesting that the 40% rate of largely positive caregiving could be a sign of resilience (cf. Masten & Narayan, 2012; Betancourt *et al.* 2015). There is evidence, indeed, that war violence can transmit to the family environment (Catani, 2010).

We included households from three diverse, conflict-prone provinces of Burundi and found some regional differences: most children who displayed antisocial behavior live in Rumonge Province. This may be due to the differences in age, as age influences behavioral expectations and possibilities for deviance (Grove, 1985). Children from Rumonge were on average 14 compared with 11 years of age in Cibitoke and Muyinga. Alternatively, the outcomes may be indicative of relatively higher levels of interpersonal violence in Rumonge compared with the other locations, for which we found some support as well. The more negative appraisal of community relations by caregivers in Rumonge supports results from other studies in conflict-affected environments which show that community context matters for the children's development (Betancourt *et al.* 2014, 2015; Cummings *et al.* 2016). As noted above (page 6), most caregivers evaluated their relations with neighbors as ‘positive’, but this appeared to signify mostly the absence of bad relations, or overt hostility. We believe that the long history of community conflict in Burundi may help explain the responses regarding positive and negative relations with neighbors.

Our study has several limitations. Firstly, our findings are based on a purposefully selected sample; we included a limited number of cases and we had selective dropout. Yet, our cases represent a great variety of vulnerable household categories from diverse corners of Burundi. Second, the study's focus was on present caregiving and resilience, meaning that we have limited insight into the influence of past experiences on our findings – a question that we will try to answer with a quantitative data set, which was collected among other households in Burundi as part of the same research partnership (Charak *et al.* 2017). For instance, we know little about the caregiver's own history of social behavior, and although we asked questions about the caregiver's care experiences during childhood, our findings were too limited to draw conclusions. Research in other contexts, however, has shown that these factors are associated with caregiving and behavior in the offspring generation (Crittenden & Ainsworth, 1989; Widom 1989; Roth *et al.* 2014; Crombach & Bambonyé, 2015). Third, due to our sampling and primary interest in caregiving and resilience, we paid limited attention to power structures in Burundian society (informed by exo and macrosystems). In view of the widespread problems of corruption, impunity (Vinck *et al.* 2015) and ongoing political crisis in Burundi, further research is needed to identify why some caregivers and children are more or less likely to experience adversity or access resources for resilience.

## Conclusions

This qualitative study with caregivers and children in vulnerable households and communities in Burundi contributes to research on caregiving and the intergenerational transmission of violence in conflict-affected environments. Findings show that in these disadvantaged socio-ecological environments, caregiving can impact children's functioning and their role in reproducing violence, both as risk and protective factors. Besides addressing structural causes of violence and intergenerational inequity (Verwimp & Bundervoet, 2009; Berckmoes & White, 2014; Laird, 2016), in line with Jordans *et al.* (2013); Rieder & Elbert (2013); and Betancourt *et al.* (2015), our findings suggest that interventions aimed at supporting caregivers are promising for breaking cycles of violence in conflict-affected environments.
